# Knowledge, Attitude, and Practice towards COVID-19 among Mothers in Dessie Town, Northeast Ethiopia, 2020

**DOI:** 10.1155/2022/4377460

**Published:** 2022-10-19

**Authors:** Debrnesh Goshiye, Zinet Abegaz, Sisay Gedamu

**Affiliations:** ^1^Department of Pediatric and Child Health Nursing, College of Medicine and Health Sciences, Wollo University, Dessie, Ethiopia; ^2^Department of Reproductive Health, College of Medicine and Health Sciences, Wollo University, Dessie, Ethiopia; ^3^Department of Comprehensive Nursing, College of Medicine and Health Sciences, Wollo University, Dessie, Ethiopia

## Abstract

**Background:**

Coronavirus disease 2019 (COVID-19) is a highly infectious disease causing a catastrophic effect, and many of us are worried to find a new normal. Many burdens are occurring in households, predominantly to women and mothers. Women worldwide are naive on COVID-19 symptoms, transmission, and prevention measures and worried on being exposed to coronavirus. The study aimed to assess the mother's knowledge, attitude, and practice towards COVID-19.

**Methods:**

A community-based cross-sectional study was conducted from July 22 to August 7, 2020, in Dessie town, Ethiopia. A total of 634 mothers were included in the study. Multistage cluster sampling was used to take the proper sample. An interviewer-administered pretested structured questionnaire was used. Data were entered into EpiData, version 3.1, and analyzed by using SPSS, version 23. Both binary and multivariate logistic regression analyses were performed to find factors associated with dependent variables. The mean score was used to grade the knowledge, attitude, and practice towards COVID-19.

**Result:**

A total of 610 mothers participated in this study, making that a 96.2% response rate. More than half of the respondents 319 (50.3%) were in the age of 28–38 years. About 438 (71.8%) attended grades 1–12. About 531 (87.5%) were married, and most respondents 399 (65.4%) were housewives. The mean scores of knowledge, attitude, and practice were 15.36 (SD = 3.059), 6.4 (1.779), and 7.38 (3.068), respectively. Moreover, about 442 (72.5%), 354 (58%), and 338 (55.4%) of the respondents had good knowledge, a favorable attitude, and good practice on COVID-19, respectively.

**Conclusion:**

Almost three-fourths of the mothers had good knowledge of COVID-19. However, more than two-fifths of the participants had an unfavorable attitude and poor practice on COVID-19 preventive measures, which may put a high risk of infection that could worsen maternal morbidity and mortality during this pandemic. Therefore, health education programs for mobilizing and improving COVID-19-related knowledge, attitude, and practice are urgently needed, especially for those mothers who have low access to information due to home duty.

## 1. Introduction

Coronavirus disease 2019 (COVID-19), which was formerly called “2019 novel coronavirus” or “2019-nCoV”, is caused by a new strain of coronavirus linked to the same family of viruses as severe acute respiratory syndrome (SARS) and some types of common cold. The disease was first reported in Wuhan, China, by the World Health Organization's (WHO) China office on December 31, 2019. This disease was started as an epidemic mainly limited to China but has now become a truly global pandemic and is declared a public health emergency of international concern (PHEIC). More than 5,595,091 people have been infected with this virus, with significant outbreaks in the US, Brazil, and Russia and over 1.1 million deaths globally [1–3].

On March 13, 2020, the Federal Ministry of Health of Ethiopia had the first confirmed COVID-19 case in Addis Ababa. As reported on October 30, 2020, there were 95, 301 confirmed cases and 1,457 deaths in the country. The pandemic has affected educational systems globally, leading to the widespread closure of schools and universities. Any age of people may be infected and transmit the virus, although older people and/or those with preexisting medical conditions seem more likely to develop serious illness [4–7].

Many of us are stressed to find a new normal during this pandemic. There is a lot of burden on households, mainly women and mothers. Across the world, even before the pandemic hit, women do more housework and childcare and have less leisure time than their male counterparts. And, as a national poll shows, women are more likely than men to have disrupted lives because of the coronavirus. The mother played an important role in all aspects of family health involving keeping the family healthy, preventing, and dealing with ill health [8, 9].

Office closures have collided with closed schools, forcing parents to work from home as they balance full-time childcare and part-time teaching. This added burden on parents is falling most heavily on mothers. Families around the globe are adapting to changes happening due to COVID-19, but many parents find it stressful to balance work, caring for children, and maintaining the household [10, 11].

The pandemic is disturbing working parents, particularly working women. About 32% of women aged 25–44 compared with 12% of men in the same age group are not working due to childcare concerns. The impacts of crises are never gender-neutral, and COVID-19 is no exception. A report shows that the pandemic will push 96 million people into extreme poverty by 2021; about half are women and girls [12, 13].

Despite the different actions taken in fighting the outbreak, the success or failure of these efforts is largely dependent on public behavior. Specifically, public adherence to preventive measures has key importance to avert the spread of the disease. Adherence is likely to be affected by the public's knowledge and attitudes towards COVID-19. Evidence shows that public knowledge is important in tackling pandemics [14, 15]. Successfully control and minimization of morbidity and mortality due to COVID-19 require changing behavior, which is influenced by people's knowledge and perceptions of the public, especially mothers [16].

By measuring public awareness and knowledge about the coronavirus, deeper visions into existing public perceptions and practices can be gained. Hence, the aim of this study was to assess mothers' knowledge, attitude, and practices (KAP) towards COVID-19 so as to identify gaps and strengthen an ongoing prevention effort. To the researchers' knowledge, this is the first study to investigate COVID-19 KAP and associated sociodemographic characteristics among mothers of Ethiopia.

## 2. Methods

### 2.1. Study Design, Period, and Setting

A community-based cross-sectional study design was conducted to assess KAP towards the COVID-19 pandemic and its associated factors among mothers from July 22 to August 7, 2020, in Dessie town, Ethiopia. Dessie town is located 401 km from Addis Ababa; 480 km from Bahir Dar. Its astronomical place is 11°38′ north latitude and 37°15′ east longitude. The town has a national total population of 382, 912. The town has 5 subcities and 18 kebeles.

### 2.2. Populations

All mothers living in Dessie town were the source population. Mothers living in the selected kebeles of Dessie town subcities were the study population. All mothers living in Dessie town were included in the study, but mothers who live in Dessie town for less than one month and those who are seriously ill were excluded.

### 2.3. Sample Size Determination

The single population proportion formula of (*n* = (*Z*_*α*/2_)^2^*P*(1 − *P*)/*d*^2^) was used. The assumptions used to calculate the sample size were as follows: 95% level of CI, which yields *Z*_*α*/2_ = 1.96, 5% margin of error, and estimated proportion (*P*) of 50% to get the greatest sample size since there was no similar study. Then, by multiplying with 1.5 for design effect and by considering a nonresponse rate of 10%, the sample size was 634.

### 2.4. Sampling Technique

Multistage cluster sampling was used to take the proper sample. Initially, Dessie town was classified into five subcities. Then, three subcities were selected by a lottery method. Then, from these randomly selected subcities, one kebele from each subcity was selected using a lottery method. The total sample size was allocated proportionally to each kebele depending on the number of households in each kebele. In each kebele, the first household was selected randomly from the central place of the kebele. The next household was selected according to the inclusion criteria based on the principle of the next nearest household. Households in the kebele were visited until the proportionally allocated sample size for each kebele was fulfilled.

### 2.5. Operational Definitions

#### 2.5.1. Knowledge

19 questions were used to assess the mothers' level of knowledge on COVID-19. The mean score for these knowledge questions was 14.38; and those mothers with a score of above the mean were considered to have adequate knowledge on COVID-19, and those scoring below the mean were considered to have inadequate knowledge on COVID-19.

#### 2.5.2. Practice

11 questions were used to assess mothers' practice on COVID-19 preventive measures. The mean score for these questions was 7.38; and women who scored above the mean of the 11 questions were considered to have adequate practice, and those who scored below the mean were considered to have inadequate practice.

#### 2.5.3. Attitude

To determine mothers' attitude towards COVID-19, eight questions were used. The mean score was 6.4; and those scoring above the mean of the eight attitude questions were considered to have a favorable attitude towards COVID-19, and those scoring below the mean were considered to have an unfavorable attitude towards COVID-19.

### 2.6. Data Collection Tool and Procedure

An interviewer-administered structured questionnaire was used to get the required data. The instrument was constructed from a review of different available studies [17–23]. It has four parts: the sociodemographic feature part, knowledge-assessing questions, attitude-assessing questions, and practice-assessing questions. Using this tool, households in selected kebeles were visited by data collectors as stated in the sampling technique until the proportionally allocated sample size in each kebele is achieved.

### 2.7. Data Analysis

Before the analysis, all filled questionnaires were checked for completeness, consistency, and accuracy. Then, the data were coded, and finally data cleaning and entry was carried out using EpiData software, version 3.1. Before analysis, the data were cleaned for inconsistencies and missing values. The analysis was performed using SPSS software, version 23. Descriptive statistics (frequency table, pie chart, and bar graph) were used to summarize the data. Bivariate and multivariate logistic regression analyses were used to check variables having a significant association with the dependent variables and to prevent for possible cofounding variables, respectively. A significant association was declared at *P* <0.05.

### 2.8. Data Quality Assurance

The questionnaire was pretested by taking 5% of the total sample size in kebeles that are not primarily selected for the study. The necessary amendments were made upon the identification of ambiguities of the tools in the wording, logic, and skipping order. The principal investigators and the supervisors checked the collected data for completeness.

### 2.9. Ethical Consideration

Ethical approval was obtained from the Research Ethical Review Committee of the College of Medicine and Health Sciences, Wollo University. An official letter of permission from the college was submitted to each kebele administrative unit to conduct the research. All the collected data were kept confidential, and no one except the members of the research team had access to the collected information. All paper and computer records of the study were kept in a secured place under lock, and the name and/or other personal information was not notified in any report.

## 3. Results

### 3.1. Sociodemographic Features

From the total calculated sample size of 634, about 610 mothers were interviewed. This makes the response rate of 96.2%. More than half of the respondents 319 (50.3%) were in the age of 28–38 years. Regarding the education status, about 438 (71.8%) attended grade 1–12. About 531 (87.5%) and 338 (55.4%) were married and Muslim in religion, respectively. Regarding occupation, most respondents were housewives, and 43.4% of them have only one child (see Table 1).

### 3.2. Availability and Accessibility of Health Care Service

About 352 (57.9%) participants have access to the health center near their home (see Table 2).

### 3.3. Knowledge about COVID-19

**Figure 1:**
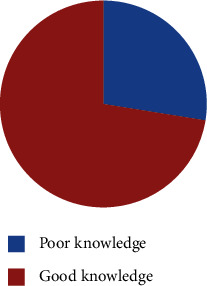
Total distribution of respondents according to their knowledge level on COVID-19 in Dessie town, Ethiopia, 2020.

Of all the respondents, 72.5% had good knowledge on COVID-19 (mean = 15.36, SD = 3.059), as shown in Figure 1. Almost all the respondents heard about the COVID-19 pandemic, and 94.3% of them knew that the disease is contiguous. About 89.8% and 92.6% of the respondents stated that cough as a symptom and admission of confirmed cases to be in an isolation center, respectively, as described in Table 3.

### 3.4. Attitude towards COVID-19

**Figure 2:**
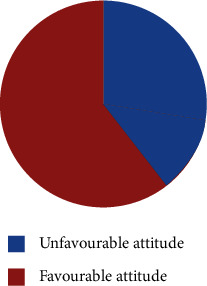
Total distribution of respondents according to their attitude towards COVID-19 in Dessie town, Ethiopia, 2020.

Of all the respondents, 354 (58.0%) had a favorable attitude towards COVID-19 (mean = 6.4, SD = 1.779), as shown in Figure 2. Most of the respondents (94.6%) provided the correct answer of maintaining distance to prevent COVID-19 spread. Only 70.5% of the respondents thought that COVID-19 can cause massive fatality in Ethiopia (Table 4).

### 3.5. Practice towards COVID-19

**Figure 3:**
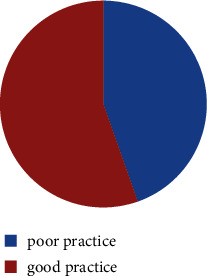
Total distribution of respondents according to their practice on COVID-19 in Dessie town, Ethiopia, 2020.

Of all the respondents, 338 (55.4%) showed good practice (mean = 7.38, SD = 3.068) on COVID-19 preventive measures, as shown in Figure 3. Most of the respondents (79.3%) provided the correct answer of sneezing between elbows to prevent COVID-19 spread, and 75.2% of the respondents stated that they frequently washed hands with a soap or sanitizer. About 80.7% of the respondents stated that they regularly deal with sick people or health workers (Table 5).

### 3.6. Factors Associated with Knowledge, Attitude, and Practice towards COVID-19 among Mothers

The determinants of knowledge towards COVID-19 among the participants are shown in Table 6. Both mother and father being the main caregivers, those using sanitizer, those with good practice, and favorable attitude were factors associated with knowledge towards COVID-19 infection among the participants. Both mother and father being the main caregivers of the family (AOR = 1.847, 95% CI: 1.136–3.004, *P*=0.013) was 3 times more likely to have adequate knowledge when compared with only mother being the main caregiver of the family. Mothers who use sanitizer (AOR = 3.159, 95% CI: 1.612–6.190, *P*=0.001) were more likely to have adequate knowledge when compared with those who do not use sanitizer. Similarly mothers with good practice (AOR = 3.059, 95% CI: 1.581–5.918, *P*=0.001) were 3 times more likely to have adequate knowledge than those with poor practice towards COVID-19 measures. And mothers with favorable attitudes (AOR = 7.763, 95% CI: 4.850–12.425, *P* < 0.001) were 7 times more likely to have adequate knowledge than those with unfavorable attitudes towards COVID-19.

Factors associated with a favorable attitude towards COVID-19 among participants are shown in Table 7. Mothers of age above 39 years (AOR = 0.06, 95% CI: 0.008–0.487, *P*=0.008) were less likely to have a favorable attitude towards COVID-19 than those younger than 27 years. Mothers educated from grade 1 to 12 (AOR = 7.180, 95% CI: 2.187–23.569, *P*=0.001) and those educated at college and above (AOR: 4.768, 95% CI: 1.388–16.372, *P*=0.013) were seven and four times more likely to have a favorable attitude towards COVID-19 when compared with mothers with no formal education, respectively. When compared with mothers having a hospital as the nearest health institution, mothers having a health center as the nearest health institution (AOR = 0.569, 95% CI: 0.338–0.956, *P*=0.033) were less likely to have a favorable attitude towards COVID-19. Similarly, mothers with the private clinic as the nearest health institution (AOR = 0.459, 95% CI: 0.249–0.847, *P*=0.013) were less likely to have a favorable attitude towards COVID-19 than mothers with a hospital as the nearest health institution. And mothers with a health post as the nearest health institution (AOR = 0.045, 95% CI: 0.005–0.438, *P*=0.008) were also less likely to have a favorable attitude towards COVID-19 than mothers with a hospital as the nearest health institution. Mothers with good knowledge about COVID-19 (AOR = 8.192, 95% CI: 5.091–13.182, *P* < 0.001) were 8 times more likely to have a favorable attitude towards COVID-19 than mothers with poor knowledge (Table 7).

Factors associated with good practice of preventive measures against COVID-19 infection are shown in Table 8. Mothers with other occupations other than a civil servant (AOR = 0.441, 95% CI: 0.260–0.746, *P*=0.002) were less likely to practice COVID-19 preventive measures than mothers being housewives. Mothers with a private clinic as the nearest health institution (AOR = 0.516, 95% CI: 0.290–0.916, *P*=0.024) were less likely to practice COVID-19 preventive measures when compared with those with a hospital as the nearest health institution. Similarly, mothers with more than 3 children (AOR = 0.516, 95% CI: 0.290, 0.916, *P*=0.024) were less likely to practice COVID-19 preventive measures than those with one child. Participants with good knowledge of COVID-19 (AOR = 4.938, 95% CI: 3.123–7.807, *P* < 0.001) were 4 times more likely to practice COVID-19 preventive measures than those with poor knowledge. And mothers with a favorable attitude towards COVID-19 (AOR = 1.691, 95% CI: 1.132–2.527, *P*=0.010) were about 2 times more likely to practice COVID-19 preventive measures than those who have an unfavorable attitude towards COVID-19 (Table 8).

## 4. Discussion

The study was intended to assess the level of knowledge, attitude, and practice of mothers on COVID-19. More than half of the respondent 319 (50.3%) were in the age of 28–38 years. About 438 (71.8%) attended grade 1–12. About 531 (87.5%) were married, and most respondents 399 (65.4%) were housewives. Moreover, about 442 (72.5%), 354 (58%), and 338 (55.4%) of the respondents had good knowledge, a favorable attitude, and good practice on COVID-19, respectively.

Based on the knowledge scores of the participants, 72% were knowledgeable about COVID-19. The result is higher than the study conducted in Kombolcha, Ethiopia, in which 58.5% are knowledgeable about COVID-19 [24]. But the result is lower than the study [25] in which about 92.7% had sufficient knowledge on COVID-19 [25]. This discrepancy may be due to the difference in the study participants in both the studies.

Our results were lower than those of previous studies regarding the KAP towards COVID-19 among residents in Nigeria [18], Iran [19], and Cameroon, which showed about 99.5%, 90%, and 84.19% of the participants were knowledgeable towards COVID-19, respectively. This difference may be because of the difference in the study population. Since our study participants were mothers, who had limited access to COVID-19 related updates (which may be posted online or media) and preventive measure. This will hurt in turn the knowledge level.

Compared with a study about KAP towards COVID-19 among pregnant women in Nigeria, which shows only 60.9% of the participants were found to be knowledgeable about COVID-19 [26], our study participants demonstrated higher knowledge about COVID-19. This discrepancy may be because pregnant mothers may be more concerned about their pregnancy-related health issues other than the pandemic.

Furthermore, 58% of the participants in this study had a favorable attitude towards COVID-19. This study result was lower than the studies conducted in India [27], Iran [19], and Cameroon [20], which shows that 97.33%, 90%, and 69% of the participants had a favorable attitude towards COVID-19, respectively. The underlying reason for these score differences could be the population and the period in which the studies were conducted. While in all these three studies, the study was done at the time of the main phase of the outbreak when the populations were exposed to a lot of information about the disease, its route transmission, and prevention ways, through which they will have a favorable attitude. On the other hand, our study is conducted among mothers who may have less information to update themselves. This result is also lower than the study conducted in Northern Ethiopia [28], which shows that about 72% of the respondents had a favorable attitude towards COVID-19. This discrepancy may be due to the difference in the study population. In Northern Ethiopia, the study was done among nurses who have easy access to information to update themselves.

The study result also shows that about 55.4% of the respondents had a good practice on COVID-19 preventive measures. This study is in line with a study conducted in Addis Zemen, Ethiopia [29]. But this study result was lower than that of the studies conducted in India [27], Iran [19], and Cameroon [20], in which 93.7%, 89%, and 60.8% of the respondents had good practice towards COVID-19 preventive measures, respectively. The possible explanation for this score difference may be the cultural and sociodemographic differences between the studies to practice preventive measures of the COVID-19 pandemic. This study result was also higher than the study conducted in Nigeria [26], which shows only 30.3% of the participants had good practice on COVID-19 preventive measures. This may be due to the difference in the study population. In Nigeria, the study populations were pregnant women who may need extra support to practice COVID-19 preventive measures.

Furthermore, our study showed that having both father and mother as the main caregivers of the family shows a significant association with good knowledge about COVID-19 (*P* = 0.013). This may be being an older aged mother who may make them inflexible and difficult to be convinced and updated compared to the youngest mothers.

On the other hand, use of sanitizer also shows a significant association with good knowledge about COVID-19 (because if the mother uses sanitizer, it is indicative to the mother knows COVID-19 preventive measures). This study also shows that good practice on COVID-19 preventive measures shows a significant association with good knowledge about COVID-19 (*P*=0.001). This indicates the fact that mothers practicing COVID-19 preventive measures do have adequate knowledge about COVID-19.

Additionally, a favorable attitude towards the COVID-19 pandemic also shows a significant association with good knowledge about the COVID-19 pandemic (*P*=0.000). This may be because a favorable attitude is a result of good knowledge of the COVID-19 pandemic.

Mothers above the age of 39 years were less likely to have a favorable attitude towards COVID-19 preventive measures (*P* = 0.008). This may be since like the age increase mothers may become difficult to convince and update their attitude; they may become more inflexible.

Mothers educated from grade 1 to 12 (*P*=0.001) and those educated from college and above (*P*=0.013) were more likely to have a favorable attitude towards COVID-19. As one gets more educated, there will be multiple ways of acquiring information to know about the prevention of COVID-19. Also, when someone gets more educated he/she will have a better understanding of control measures and preventive strategies related to COVID-19, and the attitude towards the COVID-19 pandemic will be better. Furthermore, education results in better information collection habit and lead to efficient use of health inputs for prevention of COVID-19.

Mothers with a private clinic as the nearest health institution were less likely to have a favorable attitude towards COVID-19 (*P*=0.013). This may be due to that in a private clinic, care provides may not offer any information regarding COVID-19 rather than giving other health care services, so this may affect the attitude towards COVID-19. Similarly, mothers with a health center as the nearest health institution were less likely to have a favorable attitude towards COVID-19 (*P*=0.033), since routine and limited health care services are only delivered there and some health centers are closed due to the pandemic. This may affect the attitude.

Mothers having good knowledge about COVID-19 were more likely to have a favorable attitude towards the COVID-19 pandemic (*P* < 0.001). This is because if someone had adequate knowledge about the COVID-19 pandemic, it is easy to have a positive and favorable attitude towards COVID-19 preventive measures and other related issues.

Mothers with other occupations (*P*=0.002) were less likely to practice COVID-19 preventive measures than mothers being housewives. This is because mothers with occupations other than housewives may be busy with many works and routines and may not have enough time to update themselves and to practice those COVID-19 preventive measures. Mothers with the private clinic as the nearest health institution (*P*=0.024) were also less likely to practice COVID-19 preventive measures. This may be related to the fact that the private clinic is more concerned to deal with case-related health issues. They may not offer any information related to the pandemic than treating the current health status of the patient. This further affects the practice of COVID-19 preventive measures.

Similarly, mothers with more than 3 children (*P*=0.024) were also less likely to practice COVID-19 preventive measures. It may be related with as the number of children/family members increase, the mother may be busy with many home duties and may not have enough time to update themselves. This intern will affect their practice on COVID-19 preventive measures.

On the other hand, participants with good knowledge about COVID-19 (*P* < 0.001) and with a favorable attitude towards COVID-19 (*P*=0.010) were more likely to practice COVID-19 preventive measures; this is because if someone is knowledgeable and has a favorable attitude towards COVID-19, it is easy to perform COVID-19-related preventive measures.

## 5. Conclusion

In general, about 3/4^th^ of the study participants had good knowledge of the COVID-19 pandemic. On the other hand, more than 2/5^th^ of the participants had an unfavorable attitude and poor practice on COVID-19 preventive measures. This would put at high risk of COVID-19 infection not only for mothers but also for all family members and the community in large. This would further worsen the maternal morbidity and mortality profile due to this coronavirus pandemic.

Consequently, health education platforms pointed at activating and cultivating COVID-19-related knowledge, attitude, and practice are immediately required particularly for those who have low access to information due to home duty, i.e., the mothers. Leaflets containing COVID-19 symptoms, transmission, prevention mechanism, and the like should be prepared in local languages and should be provided to the community through the home-to-home visit. And, health professionals at any health care setup should provide detailed information about COVID-19 to their customers so as to improve COVID-19-related knowledge, attitude, and practice of the mother and the community in large.

## Figures and Tables

**Table 1:** Sociodemographic characteristics of mothers in Dessie town, Ethiopia (*N* = 610).

Variable	Frequency	Percent
Main caregiver of the family		
Mother	165	27.0
Father	34	5.6
Both	401	65.7
Apart from the parents	10	1.6

Age of mother/caregiver, years		
17–27	282	46.2
28–38	319	52.3
Above 38	9	1.5

Education of mother/caregiver		
No formal education	26	4.3
Grades 1–12	438	71.8
College/university and above	146	23.9

Religion		
Orthodox	257	42.1
Muslim	338	55.4
Others	15	2.5

Marital status		
Married	531	87.0
Single	42	6.9
Widowed	19	3.1
Divorced	18	3.0

Occupation		
Housewife	399	65.4
Government worker	124	20.3
Others	87	14.3

**Table 2:** Availability and accessibility of health care service for mothers in Dessie town, Ethiopia, 2020.

Variable	Frequency	Percent
Type of health institution near their home		
Hospital	129	21.2
Health center	352	57.9
Health post	6	1.0
Private clinic	123	19.9

Time taken to reach the nearest health institution		
Less than 20 minutes	461	75.6
20 to 40 minutes	113	18.5
More than 40 minutes	36	5.9

Transportation system to health institution		
By foot	364	59.9
By car	244	40.1

**Table 3:** Knowledge of mothers about COVID-19 in Dessie, Ethiopia, 2020.

S. no.	Variable	Yes (%)	No (%)	No opinion (%)
1	Heard about the COVID-19 pandemic	609 (99.8)	1	
2	COVID-19 is contagious	575 (94.3)	34 (5.6)	1
3	COVID-19 has treatment	162 (26.6)	423 (69.3)	25 (4.09)
4	Fever is the symptom of COVID-19	537 (88.0)	68 (11.1)	5
5	Cough is the symptom of COVID-19	548 (89.8)	61 (10)	1
6	Sore throat is the symptom of COVID-19	476 (78.0)	119 (19.5)	15
7	Headache is the symptom of COVID-19	513 (84.1)	89 (14.6)	8
8	Washing hands with water and soap can eliminate the causative agent	423 (69.3)	171 (28.0)	16
9	It can be transmitted by coughing	534 (87.5)	71 (11.6)	5
10	Transmitted through contact with contaminated surfaces	516 (84.6)	80 (13.1)	14
11	Transmitted through eating meat	449 (73.6)	123 (20.2)	38 (6.22)
12	Transmitted through contact with an infected person	526 (86.2)	77 (12.6)	7
13	Health education is useful	487 (79.8)	111 (18.2)	12
14	COVID-19 can result in death	265 (43.4)	331 (54.3)	14
15	Movement should be limited	436 (71.5)	148 (24.3)	26 (4.3)
16	Markets should be closed	413 (67.7)	177 (29.0)	20
17	Schools should be closed	487 (79.8)	109 (17.9)	14
18	Suspected cases should be quarantine	555 (90.9)	43 (7.05)	12
19	Confirmed cases should be admitted to isolation centers	571 (93.6)	28 (4.59)	11

**Table 4:** Eight questions to evaluate the attitude of mothers towards COVID-19 in Dessie town, Ethiopia, 2020.

S. no.	Questions	Strongly disagree	Disagree	Neutral	Agree	Strongly agree
1	It is important to keep my distance from others	8	17	8	320	257
2	Washing hands with soap and water is essential to protect from COVID-19	6	27	9	330	238
3	Wearing a well-fitting face mask is effective in protecting us from COVID-19	21	54	13	311	211
4	I may be infected with COVID-19	29	109	29	238	205
5	Health education is helpful in protecting COVID-19	25	74	9	295	207
6	Patients with COVID-19 must be isolated	34	73	8	258	237
7	Government of Ethiopia has taken sufficient preventive measures to prevent the spread of COVID-19	96	133	14	263	104
8	COVID-19 can cause massive fatality	70	81	29	263	167

**Table 5:** Eleven questions to evaluate the level of practice on COVID-19 among mothers in Dessie town, Ethiopia, 2020.

S. No.	Questions	Response	Frequency	Percent
1	Frequently washed hands with a soap or sanitizer	No	151	24.8
		Yes	459	75.2
2	Regular use of mask	No	223	36.6
		Yes	387	63.4
3	Usually spent times in public meeting	No	203	33.3
		Yes	407	66.7
4	Maintained social distance	No	191	31.3
		Yes	419	68.7
5	Sneezed between elbows	No	126	20.7
		Yes	484	79.3
6	Frequently touched mouth or eyes or nose	No	247	40.5
		Yes	363	59.5
7	Regularly cleaned work or home or classroom table	No	215	35.2
		Yes	395	64.8
8	Usually cleaned mobile with sanitizer	No	312	51.1
		Yes	298	48.9
9	Regularly deal with sick people or health workers	No	118	19.3
		Yes	492	80.7
10	Usually shared food or water pot with others	No	268	43.9
		Yes	342	56.1
11	Often ate half-or semi cooked fish, meat, eggs, or vegetables	No	152	24.9
		Yes	458	75.1

**Table 6:** Factors associated with the knowledge of mothers about COVID-19 in Dessie town, Ethiopia, 2020.

Variable	Level of knowledge, *N* (%)		Odds ratio, at 95% CI			
	Poor (%)	Good (%)	Crude OR	*P* value	Adjusted OR	*P* value
Main caregiver of the family						
Mother	65	100	1			
Father	9	25	1.806 (0.793, 4.114)	0.160	1.659 (0.587, 4.683)	0.339
Both	94	307	2.123 (1.440, 3.131)	0.000	1.847 (1.136, 3.004)^*∗∗*^	**0.013**
Apart from parents	0	10	1050058661.791	0.999		

Educational status of mother/caregiver						
No formal education	14	12	1			
Grade 1–12	113	325	3.355 (1.507, 7.469)	0.003	1.707 (0.627, 4.651)	0.295
College and above	41	105	2.988 (1.275, 7.000)	0.012	1.521 (0.523, 4.424)	0.441

Number of children						
Having one child	65	210	1			
Having two children	57	142	0.771 (0.509, 1.167)	0.219	0.923 (0.553, 1.542)	0.760
Above 3 children	46	90	0.606 (0.386, 0.951)	0.029	0.790 (0.450, 1.388)	0.412

Use sanitizer						
No	88	63	1			
Yes	80	379	6.617 (4.420, 9.906)	0.000	3.159 (1.612, 6.190)^*∗∗*^	**0.001**

Regularly use face mask						
No	98	125	1			
Yes	70	317	3.550 (2.452, 5.140)	0.000	0.788 (0.410, 1.517)	0.476

Maintain social distance						
No	87	104	1			
Yes	81	338	3.491 (2.402, 5.074)	0.000	0.839 (0.457, 1.540)	0.571

Practice						
Poor	126	146	1			
Good	42	296	6.082 ⟶ 4.071 ⟶ 9.088	0.000	3.059 (1.581, 5.918)^*∗∗*^	**0.001**

Attitude						
Unfavorable	134	122	1			
Favorable	34	320	10.338 (6.723, 15.896)	0.000	7.763 (4.850, 12.425)^*∗∗*^	**0.000**

**Table 7:** Factors associated with the attitude of mothers towards COVID-19 in Dessie town, Ethiopia, 2020.

Variable	Attitude *N* (%)		Odds ratio, at 95% CI			
	Poor (%)	Good (%)	Crude OR	*P* value	Adjusted OR	*P* value
Age of the mother, years						
17–27	116	166	1		1	
28–38	133	186	0.977 (0.706, 1.353)	0.890	0.988 (0.671, 1.455)	0.951
Above 39	7	2	0.200 (0.041, 0.978)	0.047	0.062 (0.008, 0.487)^*∗∗*^	**0.008**

Main caregiver of the family						
Mother	86	79	1		1	
Father	13	21	1.759 (0.826, 3.746)	0.143	1.678 (0.683, 4.123)	0.259
Both	154	247	1.746 (1.211, 2.517)	0.003	1.384 (0.890, 2.152)	0.149
Apart from parents	3	7	2.540 (0.635, 10.163)	0.188	4.067 (0.589, 28.076)	0.155

Educational status						
No formal education	21	5	1		1	
Grade 1–12	170	268	6.621 (2.450, 17.892)	0.000	7.180 (2.187, 23.569)^*∗∗*^	**0.001**
College and above	65	81	5.234 (1.871, 14.638)	0.002	4.768 (1.388, 16.372)^*∗∗*^	**0.013**

Nearest health institution						
Hospital	42	87	1		1	
Health center	155	198	0.617 (0.404, 0.942)	0.025	0.569 (0.338, 0.956)^*∗∗*^	**0.033**
Health post	5	1	0.097 (0.011, 0.853)	0.035	0.045 (0.005, 0.438)^*∗∗*^	**0.008**
Private clinic	54	68	0.608 (0.364, 1.015)	0.057	0.459 (0.249, 0.847)^*∗∗*^	**0.013**

Use sanitizer						
No	98	53				
Yes	158	301	3.523 (2.395, 5.180)	0.000	1.384 (0.740, 2.589)	0.309

Regularly use face mask						
No	123	100				
Yes	133	254	2.349 (1.677, 3.291)	0.000	0.806 (0.437, 1.489)	0.491

Maintain social distance						
No	111	80				
Yes	145	274	2.622 (1.846, 3.724)	0.000	1.502 (0.837, 2.696)	0.173

Knowledge						
Poor	134	34				
Good	122	320	10.338 (6.723, 15.8960	0.000	8.192 (5.091, 13.182)^*∗∗*^	**0.000**

Practice						
Poor	154	118				
Good	102	236	3.020 (2.163, 4.216)	0.000	1.349 (0.723, 2.518)	0.346

**Table 8:** Factors associated with the practice of mothers on COVID-19 preventive measures in Dessie town, Ethiopia, 2020.

Variable	Level of practice, *N* (%)		Odds ratio, at 95% CI			
	Poor (%)	Good (%)	Crude OR	*P* value	Adjusted OR	*P* value
Occupation						
Housewife	171	228	1		1	
Civil servant	48	76	1.187 (0.786, 1.793)	0.414	1.350 (0.846, 2.153)	0.208
Others	53	34	0.481 (0.299, 0.773	0.002	0.441 (0.260, 0.746)^*∗∗*^	**0.002**

Nearest health institution						
Hospital	47	82	1		1	
Health center	161	192	0.684 (0.451, 1.035)	0.073	0.787 (0.489, 1.267)	0.324
Health post	3	3	0.573 (0.111, 2.955)	0.506	0.422 (0.076, 2.326)	0.322
Private clinic	61	61	0.573 (0.346, 0.949)	0.031	0.516 (0.290, 0.916)^*∗∗*^	**0.024**

Number of children						
Having one child	104	171	1		1	
Having two children	86	113	0.799 (0.551, 1.159)	0.237	0.941 (0.622, 1.423)	0.773
Above 3 children	82	54	0.401 (0.263, 0.610)	0.000	0.365 (0.228, 0.584)^*∗∗*^	**0.000**

Knowledge						
Poor	126	42	1		1	
Good	146	296	6.082 (4.071, 9.088)	0.000	4.938 (3.123, 7.807)^*∗∗*^	**0.000**

Attitude						
Unfavorable	154	102	1		1	
Favorable	118	236	3.020 (2.163, 4.216)	0.000	1.691 (1.132, 2.527)^*∗∗*^	**0.010**

^*∗∗*^Variables showing a significant association.

## Data Availability

The data used to support the findings of this study are included within the article.
